# Improvement in the Journal

**Published:** 1869-11

**Authors:** 


***Improvement.***

We think our readers will unite with us in the opinion that Messrs. Keen & Cooke, our new publishers, are doing excellently well their part of the work. And all concerned, editors, publishers and printers promise that the future shall show a constant advance.

In the last number were a few vexatious typographical errors, overlooked in hastily reading of proof, but hereafter biblical accuracy will be attempted. Readers will oblige us by correcting with the pen several obvious errors in some of the formulas for prescriptions given in that number under the general head, Loot.

These principally consist in confusion of the ʒ and ℥ denominations. In the September issue, the article on Chinese medicine should have been placed under a different general heading, and credited to the *California Medical Gazette.*

Please aid us with your communications.

***Specialties.***

MUCH comment is arising with regard to the more stringent enforcement of the rule against advertising of specialties, adopted by the American Medical Association. The letter of the law not infrequently seems repugnant to justice. It is a matter of notoriety that many of the most eminent members of the profession do restrict themselves to the practice of some particular specialty. They proclaim it in the colleges to which they happen to be attached, in the medical periodicals for which they write, the books they compile or edit, and, more loudly still, through partial friends in society.

It passes the comprehension of the present writer, why the briefer and more compendious mode of attaching the name of the specialty practiced to an ordinary business card or advertisement in the medical, or even secular press, should so horrify the brethren. A business card or advertisement, and a fulsome laudation of one's own skill and parade of certificates are very different affairs.

It would seem that the whole thing resolves itself to a mere question of taste, about which gentlemen may differ. To our mind, the specialist assumes the virtue of modesty, although he may have it not, when he writes, or prints his chosen function after his name, rather than, by the use of the major term only, giving space for the belief that he is ready for business in any department.

To the writer it appears that the real point is: Does the avowed specialist *know* any more about it than "any other man"?

***Ozone.***

HAVE the physicians of this section noticed the remarkable abundance of ozone in the atmosphere during the past six weeks? The unusual prevalence of catarrhal disorders directed our own attention to the subject, and almost daily observations have shown

